# 

**Published:** 1909-09

**Authors:** 


					﻿Sixty4ifth Year.
Vol. LXV.
SEPTEMBER, 1909.
No. 2
BUFFALO 2
MEDICALJOURNAL
1845 by AUSTIN FLINT M. D.
"	EDITOR:
WILLIAM WARREN POTTER, M. D.
ASSISTANT EDITORS:
WILLIAM C. KRAUSS, M. D.	NELSON W. WILSON, M. D.
ASSOCIATE EDITORS:
JAMES WRIGHT PUTNAM, M. D. ERNEST WENDE, M.D.
JOHN PARMENTER, M. D.	JOHN A. MILLER, Ph. D.
MAUD J. FRYE, M. D.
				

